# Monitoring, Control, and Clinical Outcomes Associated With Chronic Kidney Disease-Mineral Bone Disorder: A Population-Based Cohort Study in Ontario, Canada

**DOI:** 10.1016/j.xkme.2025.101080

**Published:** 2025-08-06

**Authors:** Akshay Varghese, Yuguang Kang, Andrea Cowan, Rachel Holden, Ron Wald, Kristin K. Clemens

**Affiliations:** 1 Department of Medicine, Schulich School of Medicine and Dentistry, Western University, London, ON, Canada; 2 ICES, ON, Canada; 3 Department of Medicine, Division of Nephrology, Western University, London, Ontario, Canada; 4 London Health Sciences Centre, London, ON, Canada; 5 School of Medicine, Division of Nephrology, Queen’s University, Kingston, ON, Canada; 6 Division of Nephrology, Department of Medicine, University of Toronto, Toronto, ON, Canada; 7 St Michael’s Hospital, Toronto, ON, Canada; 8 Department of Medicine, Division of Endocrinology and Metabolism, Western University, London, Ontario, Canada; 9 Centre for Diabetes, Endocrinology and Metabolism, St Joseph’s Health Care London, London, ON, Canada; 10 Lawson Research Institute, London, ON, Canada

**Keywords:** Chronic kidney disease, dialysis, phosphate, parathyroid hormone, calcium, alkaline phosphatase

## Abstract

**Rationale & Objective:**

Chronic kidney disease-mineral and bone disorder (CKD-MBD) affects bone and cardiovascular health. We examined the monitoring, control, and outcomes associated with CKD-MBD.

**Study Design:**

Observational cohort study using ICES administrative data.

**Setting& Participants:**

Adults aged 40 years and older from Ontario, Canada, with at least 2 outpatient estimated glomerular filtration rate values or receiving dialysis between January 2017 and March 2020.

**Exposure:**

CKD stage based on the estimated glomerular filtration rate.

**Outcomes:**

Albumin-corrected serum calcium, phosphate, alkaline phosphatase, parathyroid hormone, and 25 hydroxyvitamin D testing and control at 365 days, and the percentage of patients monitored and controlled per guidelines. We also examined the association between laboratory values, fragility fracture, and major adverse cardiovascular events (MACE) in CKD stage 4, 5 and dialysis.

**Analytical Approach:**

Descriptive statistics were used for primary and secondary outcomes. For exploratory outcomes, we examined the cumulative incidence and incidence rate of fragility fracture and MACE based on laboratory values, and adjusted analyses using multivariable Cox proportional hazards models.

**Results:**

There were 2,580,781 people included, of whom, 303,884 had CKD (stage 3A or higher). Monitoring and control of CKD-MBD was suboptimal across the CKD spectrum. Even in maintenance dialysis, the proportion who met laboratory monitoring targets was low (5.1% had all tests measured over 365 days). The most commonly controlled laboratory value was alkaline phosphatase (55.6% were at target across the CKD spectrum). In exploratory analysis, a small protective effect of a higher calcium and phosphate level on fragility fracture was observed, with a parathyroid hormone level of 20-80 pmol/L appearing optimal for bone health in dialysis. There appeared to be a small statistically significant association between higher levels of alkaline phosphatase and phosphate with MACE in dialysis.

**Limitations:**

Results are only generalizable to adults with laboratory tests reported within the Ontario Laboratory Information System. Exploratory analyses were limited by events.

**Conclusions:**

There are gaps in the monitoring and control of CKD-MBD in Ontario, even in groups in which evidence to support management is highest. Focused studies on whether the control of CKD-MBD improves patient-important outcomes remain important.

## Plain-Language Summary

Calcium, phosphate, parathyroid hormone, and vitamin D are important for to kidney health. We examined how often these tests are being measured in people with kidney disease and how well they are being controlled in the real world. We found several gaps in the measurement and control of these laboratory tests - it does appear that having laboratory values in a good range is important for both bone and heart health based on our study. We hope that this study helps doctors and patients, and that it promotes more research in this theme.

Declining kidney function has been well associated with hyperphosphatemia, hypocalcemia, reduced activation of 25-hydroxyvitamin D (25 OHD), and secondary hyperparathyroidism (parathyroid hormone [PTH]). The term chronic kidney disease-mineral bone disorder (CKD-MBD) encompasses these metabolic changes, as well as their known effects on vascular and bone health.^1^ It is known for example that hyperparathyroidism contributes to reduced bone density and quality, and can increase fracture risk in people with CKD by 2-fold, compared with the general population.^2^ Both high and low levels of serum PTH have been associated with mortality in observational studies of patients receiving dialysis.^3^ Hyperphosphatemia and hypercalcemia contribute to vascular calcification and endothelial dysfunction, which are associated with major adverse cardiovascular events (MACE).^4^^,^^5^

The 2017 Kidney Disease: Improving Global Outcomes guidelines (KDIGO) recommend that clinicians monitor calcium, phosphate, and PTH every 6-12 months in CKD stage 3 and 4 (ie, estimated glomerular filtration rate [eGFR] 15-60 mL/min/1.73 m^2^), and even more frequently in those with end-stage kidney disease (including dialysis) (ie, every 1-3 months).^6^^,^^7^ As alkaline phosphatase (ALP) and 25 OHD have important roles in bone mineralization, KDIGO also recommends annual measurement of both indices. Although the control (ie, targets) of these markers remains controversial in the predialysis population, in those receiving dialysis, a PTH value 2-9 times the upper limit of normal for the PTH assay used is generally well accepted, with phosphate and calcium values toward normal values. The target range for 25 OHD (and use of nutritional vitamin D replacement [ie, D2/D3] remains a subject of debate).^8^ Most experts agree that deficiency (ie, a 25 OHD level of <20 or <30 mmol/L) could be harmful, but there are no devoted studies of optimal levels in CKD populations. Experts recommend vitamin D per the general population, but others suggest use only in those with hyperparathyroidism who do not have hyperphosphatemia or hypercalcemia (and then titrating doses based on PTH).^9^^,^^10^

An understanding of current practices in the monitoring, control, and clinical outcomes associated with CKD-MBD in the real world is important. Studies can help identify clinical gaps, generate data about the potential clinical consequences of CKD-MBD (eg, fracture), and inform new research in this theme (eg, sample size calculations). Prior studies are dated, have been mostly cross-sectional, and have been limited in sample size (fewer than 200 patients).^11^ Few studies have examined the association between control of laboratory values and important clinical outcomes including fragility fractures and MACE, particularly in predialysis CKD. We aimed to examine the monitoring, control, and outcomes associated with CKD-MBD in a large cohort of real-world Canadian patients.

## Methods

### Study Design and Setting

We conducted a population-based cohort study in Ontario (Canada’s most populous province), between January 1, 2017, and March 31, 2022. We report our study according to the Reporting of Studies Conducted Using Observational Routinely-Collected Health Data statement (Table S1).^12^

Residents of Ontario have access to “universal” health care including physician services, diagnostic testing, hospitals, and outpatient care. Prescription medication coverage is available to those aged 65 years and older or on social assistance, through the Ontario Drug Benefits program. Information on use of health services is maintained in linked administrative health databases held at ICES. The use of data in this project was authorized under Section 45 of Ontario’s Personal Health Information Protection Act, and the Research Ethics Board did not require additional review.

### Population

We included adults aged 40 years or older, who were either receiving maintenance dialysis (hemodialysis [HD] or peritoneal dialysis) or had evidence of at least 2 outpatient eGFR values separated by more than 90 days, but less than 730 days and within 5 mL/min/1.73 m^2^, or 5% of each other between January 1, 2017, and March 31, 2020. We limited to those aged 40 years and older as the risk of both fragility fracture and MACE increases beyond this age.^13^

After standard data cleaning (ie, excluding records with invalid or missing identification numbers that precluded linkage, missing age or sex, death on or before the index date, or non-Ontario residents), we excluded: (1) those who lived outside the catchment of laboratories who contribute values to the Ontario Lab Information System (to ensure that we could present all laboratory testing for those in data set)^14^ and (2) those with a history of kidney transplant within 365 days before the index date (as their care is guided by different clinical practice guidelines, and the use of immunosuppressive medications can affect fracture outcomes). We then categorized patients into CKD stages as defined by KDIGO. If a patient met both eGFR and dialysis criteria for inclusion, they were preferentially grouped in our dialysis cohort. Patients without CKD (ie, eGFR ≥ 60 mL/min/1.73 m^2^) served as a referent population. Unique patients could be included only once using the earliest eligible creatinine over the study period.

### Data Sources and Definitions

Patient characteristics, comorbid conditions, use of prescription medications, health care utilization, and outcomes were obtained from the ICES administrative data sets described in Table S2. Data sets included the Canadian Institute of Health Information’s Discharge Abstract Database, the National Ambulatory Care System database (emergency department visits), the Ontario Health Insurance Plan database, and the Drug Identification Number database. Data sets were linked using unique coded identifiers and analyzed at ICES.

A description of study variables is provided in Table S3. We present baseline demographics and comorbid conditions in 5 years before the index date, baseline health care use and laboratory testing in the preceding 365 days, and medication usage (where available) in the preceding 180 days.

### Outcomes

The start time for follow-up (ie, the index date) was the date of the patient’s second eligible eGFR (or the date on which maintenance dialysis was initiated). Our primary outcome(s) was the “monitoring” of laboratory tests over 365 days based on CKD stage (ie, the number of serum albumin--corrected calcium, phosphate, ALP, PTH, and 25 OHD tests per patient over 365 days). We chose a 365-day follow-up period given kidney function can decline over time. We also calculated the percentage of patients with CKD who met monitoring targets per KDIGO guidelines (Table S4).

Secondary outcomes focused on the control of CKD-MBD over 365 days based on CKD stage. Given that targets are most established in those receiving maintenance dialysis, we a priori focused on this subgroup. Given the known variability of PTH values based on laboratory, we also defined a target of 2X-9X the upper limit of normal for the laboratory test center that the patient visited (consistent with the KDIGO guidelines).

As prespecified exploratory outcomes, we extended follow-up to 2 years to examine the following potential consequences of CKD-MBD: (1) fragility fracture and (2) MACE. We used validated coding algorithms to examine fragility fracture and MACE; algorithms perform well and have been used across several ICES research studies (Table S3).^15^^,^^16^

### Statistical Analysis

We used descriptive statistics to describe baseline characteristics by CKD stage, as well as the monitoring and control of laboratory tests of interest. We reported numbers and percentages, means (standard deviations), and medians (interquartile range). If a unique patient had multiple laboratory values available over the follow-up period (eg, 3 or more calcium tests within 365 days), we defined patients as “at-target” if most of their measures were in range (eg, three-fourth measures in range). As KDIGO guidelines do not promote a definitive phosphate target in maintenance dialysis, we used a prespecified cutoff value of 1.8 mmol/L.

To examine the association between the control of measured laboratory tests, MACE, and fragility fracture, we calculated the cumulative incidence and incidence rate of these outcomes. For these analyses, we used the patients’ last eligible creatinine level available within the accrual period as our start time for follow-up. We chose to censor exploratory outcomes at 2 years because CKD stage can progress with time and impact these outcomes. We used multivariable Cox proportional hazards models to examine the independent association between laboratory values and MACE/fragility fracture, adjusted for age, sex, phosphate, calcium, PTH, ALP, and CKD stage. Calcium and phosphate were modeled per 0.1 mmol/L (1.8 mg/dL), PTH was modeled per 10 pmol/L (95 pg/mL), and ALP was modeled per 10 U/L (0.167 ukat/L). We also modeled relationships using clinically relevant changes in calcium and phosphate as post hoc analyses (ie, calcium and phosphate per 0.5 mmol/L or 2 mg/dL). We included an interaction term for CKD stage in each model and assessed for linearity in covariates using restricted cubic splines.

Given that a U-shaped relationship between PTH and fragility fracture has been observed in prior observational studies,^17^^,^^18^ as a post hoc analysis, we examined for this relationship using a restricted cubic spline function with 5 knots placed at 5th, 25th, 50th, 75th, and 95th percentiles. A PTH of 40 pmol/L (377.2 pg/mL) was our referent. We conducted in 2 groups - CKD 4-5 and dialysis. We considered a 2-tailed *P* value of <0.05 as statistically significant. *P* values were not adjusted for multiple statistical testing. We performed all analyses using SAS version 9.4 (SAS Institute).

## Results

There were 2,580,781 patients included in our cohort, of whom 303,884 had CKD 3A or higher. The mean age of patients across the CKD spectrum ranged from 55.7-75.5 years and 46.1% were men. The most common comorbid conditions were hypertension and diabetes (Table 1).

**Table 1:** Baseline Characteristics of Included Patients Based on CKD Stage

Characteristic		Stage 1	Stage 2	Stage 3A	Stage 3B	Stage 4	Stage 5	Dialysis
		N = 1,215,150	N = 1,061,747	N = 191,761	N = 76,452	N = 20,699	N = 3,765	N = 11,207
Age (y)	Mean (SD)	55.67 (9.81)	66.16 (11.82)	75.50 (10.53)	78.82 (10.49)	78.79 (11.60)	71.32 (14.17)	67.08 (12.36)
	Median (Q1-Q3)	55.00 (48.00-63.00)	66.00 (58.00-75.00)	76.00 (69.00-83.00)	80.00 (73.00-86.00)	81.00 (72.00-87.00)	73.00 (62.00-82.00)	68.00 (58.00-77.00)
	40-59	790,411 (65.0%)	317,508 (29.9%)	14,627 (7.6%)	3,965 (5.2%)	1,470 (7.1%)	817 (21.7%)	3,173 (28.3%)
	60-79	417,604 (34.4%)	595,171 (56.1%)	104,159 (54.3%)	31,904 (41.7%)	7,963 (38.5%)	1,692 (44.9%)	6,059 (54.1%)
	80+	7,135 (0.6%)	149,068 (14.0%)	72,975 (38.1%)	40,583 (53.1%)	11,266 (54.4%)	1,256 (33.4%)	1,975 (17.6%)
Sex	Female	673,302 (55.4%)	549,486 (51.8%)	107,096 (55.8%)	44,083 (57.7%)	11,671 (56.4%)	1,752 (46.5%)	4,174 (37.2%)
	Male	541,848 (44.6%)	512,261 (48.2%)	84,665 (44.2%)	32,369 (42.3%)	9,028 (43.6%)	2,013 (53.5%)	7,033 (62.8%)
Charlson	Mean (SD)	0.12 (0.63)	0.16 (0.71)	0.36 (1.04)	0.68 (1.42)	1.15 (1.79)	2.11 (2.21)	2.07 (2.13)
	Median (Q1-Q3)	0.00 (0.00-0.00)	0.00 (0.00-0.00)	0.00 (0.00-0.00)	0.00 (0.00-1.00)	0.00 (0.00-2.00)	2.00 (0.00-4.00)	2.00 (0.00-3.00)
HTN	n (%)	494,683 (40.7)	622,883 (58.7)	156,930 (81.8)	69,378 (90.7)	19,231 (92.9)	3,530 (93.8)	10,383 (92.6)
DM	n (%)	310,017 (25.5)	272,365 (25.7)	71,342 (37.2)	36,521 (47.8)	11,648 (56.3)	2,280 (60.6)	7,361 (65.7)
Prior transplant	n (%)	250 (0.0)	1,034 (0.1)	714 (0.4)	560 (0.7)	220 (1.1)	144 (3.8)	538 (4.8)
Obesity	n (%)	13,569 (1.1)	9,374 (0.9)	2,632 (1.4)	1,475 (1.9)	505 (2.4)	129 (3.4)	526 (4.7)
CAD	n (%)	103,517 (8.5)	155,162 (14.6)	45,521 (23.7)	22,932 (30.0)	6,925 (33.5)	1,508 (40.1)	4,534 (40.5)
PAD	n (%)	3,556 (0.3)	6,220 (0.6)	2,722 (1.4)	1,647 (2.2)	574 (2.8)	354 (9.4)	1,039 (9.3)
CVD	n (%)	39,271 (3.2)	63,700 (6.0)	20,189 (10.5)	10,297 (13.5)	3,138 (15.2)	708 (18.8)	1,840 (16.4)
Liver disease	n (%)	89,446 (7.4)	54,326 (5.1)	8,497 (4.4)	3,564 (4.7)	1,096 (5.3)	350 (9.3)	1,470 (13.1)
Depression	n (%)	114,307 (9.4)	92,947 (8.8)	16,857 (8.8)	6,776 (8.9)	1,864 (9.0)	473 (12.6)	1,257 (11.2)
RA	n (%)	25,863 (2.1)	25,364 (2.4)	5,688 (3.0)	2,446 (3.2)	684 (3.3)	97 (2.6)	328 (2.9)
Psoriasis	n (%)	31,412 (2.6)	25,589 (2.4)	4,258 (2.2)	1,584 (2.1)	446 (2.2)	69 (1.8)	276 (2.5)
OA	n (%)	43,793 (3.6)	61,652 (5.8)	15,761 (8.2)	7,050 (9.2)	1,775 (8.6)	291 (7.7)	769 (6.9)
COPD	n (%)	132,157 (10.9)	162,811 (15.3)	43,640 (22.8)	21,197 (27.7)	6,134 (29.6)	998 (26.5)	2,997 (26.7)
Asthma	n (%)	171,525 (14.1)	155,502 (14.6)	29,735 (15.5)	12,323 (16.1)	3,333 (16.1)	552 (14.7)	1,754 (15.7)
IBD	n (%)	8,416 (0.7)	6,319 (0.6)	896 (0.5)	354 (0.5)	95 (0.5)	19 (0.5)	46 (0.4)
SLE	n (%)	481 (0.0)	409 (0.0)	101 (0.1)	57 (0.1)	21 (0.1)	8 (0.2)	50 (0.4)
Major cancer	n (%)	97,529 (8.0)	119,275 (11.2)	26,928 (14.0)	11,588 (15.2)	3,239 (15.6)	599 (15.9)	1,845 (16.5)
Dementia	n (%)	10,709 (0.9)	38,713 (3.6)	16,292 (8.5)	8,901 (11.6)	2,717 (13.1)	480 (12.7)	622 (5.6)
Parathyroidectomy	n (%)	755 (0.1)	957 (0.1)	329 (0.2)	172 (0.2)	70 (0.3)	83 (2.2)	198 (1.8)
Nephrologist visit	Mean (SD)	0.03 (0.35)	0.05 (0.56)	0.21 (1.10)	0.68 (2.21)	2.01 (4.53)	7.27 (15.72)	10.13 (15.05)
	Median (Q1-Q3)	0.00 (0.00-0.00)	0.00 (0.00-0.00)	0.00 (0.00-0.00)	0.00 (0.00-1.00)	1.00 (0.00-3.00)	3.00 (0.00-7.00)	6.00 (2.00-12.00)
Endocrinologist visit	Mean (SD)	0.20 (1.05)	0.19 (1.03)	0.30 (1.39)	0.47 (1.88)	0.71 (2.40)	0.83 (2.64)	1.59 (4.59)
	Median (Q1-Q3)	0.00 (0.00-0.00)	0.00 (0.00-0.00)	0.00 (0.00-0.00)	0.00 (0.00-0.00)	0.00 (0.00-0.00)	0.00 (0.00-0.00)	0.00 (0.00-1.00)
Oral steroids	n (%)	11,990 (1.0)	27,231 (2.6)	9,282 (4.8)	5,035 (6.6)	1,590 (7.7)	229 (6.1)	820 (7.3)
Opioids	n (%)	31,364 (2.6)	68,492 (6.5)	22,535 (11.8)	11,271 (14.7)	3,228 (15.6)	614 (16.3)	1,792 (16.0)
Calcitonin	n (%)	1-5^a^	22 (0.0)	6 (0.0)	1-5^a^	0 (0.0)	0 (0.0)	1-5^a^
Bisphosphonates	n (%)	21,964 (1.8)	55,742 (5.3)	17,448 (9.1)	7,662 (10.0)	1,447 (7.0)	71 (1.9)	194 (1.7)
Denosumab	n (%)	5,469 (0.5)	16,817 (1.6)	5,168 (2.7)	2,302 (3.0)	548 (2.6)	42 (1.1)	94 (0.8)
SERMs	n (%)	383 (0.0)	932 (0.1)	280 (0.1)	112 (0.1)	27 (0.1)	1-5^a^	1-5^a^
Teriparatide	n (%)	1-5^a^	18 (0.0)	1-5^a^	0 (0.0)	1-5^a^	0 (0.0)	1-5^a^
Metformin	n (%)	51,178 (4.2)	102,116 (9.6)	34,413 (17.9)	14,713 (19.2)	2,020 (9.8)	50 (1.3)	372 (3.3)
DPP4 inhibitor	n (%)	22,621 (1.9)	46,165 (4.3)	18,285 (9.5)	11,253 (14.7)	3,796 (18.3)	409 (10.9)	1,409 (12.6)
Sulfonylurea	n (%)	15,393 (1.3)	30,733 (2.9)	12,904 (6.7)	7,903 (10.3)	2,464 (11.9)	203 (5.4)	598 (5.3)
GLP-1 agonist	n (%)	21 (0.0)	40 (0.0)	22 (0.0)	13 (0.0)	1-5^a^	1-5^a^	1-5^a^
SGLT2i	n (%)	9,103 (0.7)	15,915 (1.5)	4,240 (2.2)	1,173 (1.5)	112 (0.5)	0 (0.0)	60 (0.5)
Thiazolidinedione	n (%)	123 (0.0)	422 (0.0)	197 (0.1)	131 (0.2)	33 (0.2)	1-5^a^	8 (0.1)
Meglitinide	n (%)	14 (0.0)	40 (0.0)	39 (0.0)	44 (0.1)	47 (0.2)	15 (0.4)	29 (0.3)
Acarbose	n (%)	546 (0.0)	1,092 (0.1)	449 (0.2)	310 (0.4)	79 (0.4)	1-5^a^	13 (0.1)
Insulin	n (%)	10,059 (0.8)	21,965 (2.1)	11,094 (5.8)	8,791 (11.5)	4,010 (19.4)	710 (18.9)	2,212 (19.7)
ACEi/ARB	n (%)	108,921 (9.0)	293,249 (27.6)	102,461 (53.4)	47,449 (62.1)	11,340 (54.8)	1,076 (28.6)	3,037 (27.1)
CCB	n (%)	52,936 (4.4)	144,047 (13.6)	53,446 (27.9)	29,016 (38.0)	9,600 (46.4)	1,461 (38.8)	4,153 (37.1)
Diuretics	n (%)	33,837 (2.8)	107,976 (10.2)	49,385 (25.8)	30,450 (39.8)	10,489 (50.7)	1,253 (33.3)	3,928 (35.0)
Alpha blocker	n (%)	2,659 (0.2)	9,499 (0.9)	4,362 (2.3)	3,305 (4.3)	1,800 (8.7)	372 (9.9)	1,567 (14.0)
Vasodilator	n (%)	249 (0.0)	1,016 (0.1)	835 (0.4)	1,140 (1.5)	971 (4.7)	272 (7.2)	880 (7.9)
Central agonists	n (%)	474 (0.0)	1,181 (0.1)	500 (0.3)	348 (0.5)	189 (0.9)	51 (1.4)	203 (1.8)
Statin	n (%)	116,246 (9.6)	305,216 (28.7)	95,190 (49.6)	44,035 (57.6)	12,208 (59.0)	1,709 (45.4)	4,575 (40.8)
No. of unique medications	Mean (SD)	1.09 (2.88)	3.30 (4.34)	6.43 (5.00)	8.28 (5.34)	9.35 (5.93)	8.27 (7.13)	7.63 (7.77)
	Median (Q1-Q3)	0.00 (0.00-0.00)	1.00 (0.00-6.00)	6.00 (3.00-9.00)	8.00 (5.00-11.00)	9.00 (6.00-13.00)	9.00 (0.00-13.00)	7.00 (0.00-14.00)
	0	988,608 (81.4%)	504,455 (47.5%)	32,235 (16.8%)	8,046 (10.5%)	2,593 (12.5%)	1,177 (31.3%)	4,696 (41.9%)
	1-2	48,461 (4.0%)	97,941 (9.2%)	14,775 (7.7%)	2,861 (3.7%)	451 (2.2%)	47 (1.2%)	113 (1.0%)
	3-4	52,876 (4.4%)	123,144 (11.6%)	25,943 (13.5%)	7,222 (9.4%)	1,142 (5.5%)	108 (2.9%)	205 (1.8%)
	5-6	45,085 (3.7%)	113,364 (10.7%)	31,350 (16.3%)	11,302 (14.8%)	2,216 (10.7%)	225 (6.0%)	419 (3.7%)
	7-8	31,582 (2.6%)	85,250 (8.0%)	28,439 (14.8%)	12,358 (16.2%)	2,819 (13.6%)	311 (8.3%)	595 (5.3%)
	9+	48,538 (4.0%)	137,593 (13.0%)	59,019 (30.8%)	34,663 (45.3%)	11,478 (55.5%)	1,897 (50.4%)	5,179 (46.2%)
Laboratory testing in the year prior								
Ca^2+^	Mean (SD)	2.23 (0.30)	2.25 (0.29)	2.29 (0.27)	2.31 (0.23)	2.30 (0.23)	2.25 (0.33)	2.24 (0.33)
	Median (Q1-Q3)	2.29 (2.22-2.36)	2.31 (2.24-2.38)	2.33 (2.26-2.40)	2.33 (2.26-2.40)	2.32 (2.24-2.40)	2.31 (2.19-2.42)	2.28 (2.15-2.41)
	Missing	1,127,928 (92.8%)	961,998 (90.6%)	160,346 (83.6%)	52,668 (68.9%)	7,572 (36.6%)	250 (6.6%)	1,351 (12.1%)
PO^43-^	Mean (SD)	1.12 (0.19)	1.11 (0.19)	1.10 (0.19)	1.13 (0.19)	1.21 (0.22)	1.56 (0.45)	1.72 (0.53)
	Median (Q1-Q3)	1.12 (1.00-1.24)	1.11 (0.99-1.23)	1.10 (0.98-1.23)	1.13 (1.01-1.26)	1.20 (1.07-1.33)	1.49 (1.26-1.78)	1.65 (1.37-2.01)
	Missing	1,157,471 (95.3%)	997,867 (94.0%)	168,827 (88.0%)	55,569 (72.7%)	7,993 (38.6%)	270 (7.2%)	1,505 (13.4%)
ALP	Mean (SD)	77.37 (42.90)	75.73 (35.95)	79.13 (41.87)	83.09 (44.70)	89.28 (45.85)	107.46 (76.81)	110.13 (94.93)
	Median (Q1-Q3)	71.00 (59.00-87.00)	71.00 (58.00-86.00)	73.00 (59.00-89.00)	76.00 (61.00-94.00)	81.00 (65.00-102.00)	89.00 (69.00-120.00)	88.00 (68.00-121.00)
	Missing	848,073 (69.8%)	711,091 (67.0%)	119,964 (62.6%)	44,563 (58.3%)	8,852 (42.8%)	507 (13.5%)	2,042 (18.2%)
PTH	Mean (SD)	5.13 (3.13)	5.70 (3.94)	6.72 (4.63)	8.30 (6.80)	13.11 (13.42)	43.75 (50.03)	43.67 (49.10)
	Median (Q1-Q3)	4.50 (3.40-6.00)	4.90 (3.60-6.70)	5.70 (4.10-8.10)	6.90 (4.80-10.00)	10.20 (6.70-15.90)	28.70 (14.40-54.20)	30.40 (15.80-52.80)
	Missing	1,196,073 (98.4%)	1,040,023 (98.0%)	182,529 (95.2%)	65,941 (86.3%)	13,011 (62.9%)	744 (19.8%)	2,741 (24.5%)
25 OHD	Mean (SD)	75.07 (36.24)	83.01 (35.81)	83.65 (35.56)	81.47 (33.26)	77.27 (34.77)	67.41 (34.75)	63.96 (37.99)
	Median (Q1-Q3)	71.00 (52.00-92.00)	79.00 (61.00-100.00)	81.00 (62.00-101.00)	80.00 (60.00-100.00)	76.00 (55.00-96.00)	63.00 (39.00-87.00)	60.00 (35.00-85.00)
	Missing	1,144,306 (94.2%)	990,756 (93.3%)	177,264 (92.4%)	69,419 (90.8%)	18,142 (87.6%)	3,390 (90.0%)	9,675 (86.3%)

Abbreviations: ACEi, angiotensin-converting enzyme inhibitors; ALP, alkaline phosphatase; ARB, angiotensin receptor blocker; CAD, coronary artery disease; CCB, calcium channel blocker; CKD, chronic kidney disease; COPD, chronic obstructive pulmonary disease; CVD, cardiovascular disease; DM, diabetes mellitus; DPP4, dipeptidyl peptidase-4; GLP-1, glucagon-like peptide-1; HTN, hypertension; IBD, inflammatory bowel disease; OA, osteoarthritis; 25 OHD, 25-hydroxyvitamin D; PAD, peripheral artery disease; PTH, parathyroid hormone; RA, rheumatoid arthritis; SD, standard deviation; SERM, selective estrogen receptor modulator; SGLT2i, sodium-glucose cotransporter 2 inhibitors; SLE, systemic lupus erythematosus.

a Cell sizes <6 are not presented (to adhere with privacy policy).

**Figure 1:**
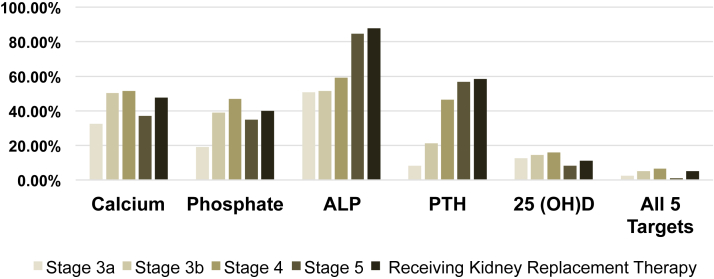
Percentage of patients who met laboratory monitoring targets based on CKD stage. Percentage of patients who met monitoring targets per KDIGO over 365 days of follow-up. ALP, alkaline phosphatase; CKD, chronic kidney disease; KDIGO, Kidney Disease: Improving Global Outcomes guidelines; 25 OHD, 25-hydroxyvitamin D; PTH, parathyroid hormone.

The “monitoring” of calcium, phosphate, ALP, PTH, and 25 OHD over 365 days is provided in Fig 1. In those with at least 1 laboratory test available, the mean and median number of laboratory tests appeared highest in the dialysis population, except for 25 OHD (Table S5). Where the percentage of patients meeting monitoring targets was examined based on CKD stage (Fig 1), only half of those receiving dialysis met calcium monitoring targets, 40.0% met phosphate targets, 58.5% met PTH targets, and 11.1% met 25 OHD targets. Just over 5% met all 5 targets (ie, calcium, phosphate, ALP, PTH, and 25 OHD) in dialysis. Laboratory monitoring was even lower in CKD 5 with the percentage meeting all 5 targets that were <5%.

Real-world “Control” of CKD-MBD (ie, laboratory values) is illustrated in Table S6. In patients with at least 1 laboratory value available, phosphate, ALP, and PTH levels were higher as kidney disease advanced. Albumin-corrected serum calcium and 25 OHD values were on the other hand, lower. Fewer than 1% of patients receiving dialysis had all laboratory tests in target. Where 25 OHD was excluded (as there remains controversy about routine testing) this increased to 13.1% of patients (Table S7).

The cumulative incidence of fracture and MACE over 2 years is provided in Table S8. The risk of both fragility fracture and MACE was highest among people receiving maintenance dialysis (40.2 per 1,000 person years and 368.5 per 1,000 person years, respectively).

Age- and sex-adjusted associations between serum calcium, PTH and ALP, fragility fracture, and MACE in CKD stage 4, 5 and dialysis are provided in Tables S9 and S10. Although outcome events were limited, there appeared to be a protective relationship between higher serum calcium, higher phosphate, and fragility fracture (hazard ratio 0.97, 0.95-0.99 and hazard ratio 0.98, 0.96-0.99), with no interaction by CKD stage (*P* = 0.19 and *P* = 0.12, respectively). Higher ALP appeared to associate with fragility fracture (HR 1.02, 1.01-1.02), with no interaction with CKD stage (*P* = 0.91).

Higher serum phosphate was associated with a heightened risk of MACE across all CKD stages with no interaction by CKD stage (*P* = 0.07). Similar findings were observed in the relationship between ALP and MACE in CKD stage 4 and dialysis. Owing to a low number of 25 OHD values, we were unable to do any meaningful analysis with this measure.

The relationship between PTH and fragility fracture in stage 4-5 CKD and dialsyis is provided in Figs S1 and S2. There was a U-shaped relationship in the association between PTH and fragility fracture in dialysis (Fig S2). Compared with patients with a PTH level of 40 pmol/L or 377.2 pg/mL (reference level), those with a PTH level between 20 and 40 pmol/L (188.6-377.2 pg/mL) had a lower fracture hazard. When the PTH level increased to 80 pmol/L (754.4 pg/mL), fracture risk surpassed the reference risk.

## Discussion

In this large population-based cohort study, we provide a contemporary update on the monitoring, control, and potential consequences of CKD-MBD in adults aged 40 years and older with CKD. Despite the presence of clinical practice guidelines, there appeared to be gaps in the monitoring and management of CKD-MBD, even in people receiving dialysis (highly monitored, most evidence to support testing). The very low number of patients who received 25 OHD testing is likely due to a lack of randomized controlled trial evidence to support use of nutritional vitamin D in advanced CKD and dialysis. Whereas some observational studies suggest a benefit of nutritional vitamin D on lowering PTH, there have been no studies on whether supplementation reduces fracture outcomes.^8^ The Survival Improvement with Cholecalciferol in Patients on Dialysis Registry Trial will examine whether cholecalciferol increases survival in UK dialysis patients and may provide more insights into the benefits/risks of supplementation.^19^

The results of our exploratory analyses, although limited by sample size, warrant discussion. Consistent with previous studies, we did find a modest relationship between phosphate and MACE. Ganesh et al^20^ also observed an increased risk of coronary artery disease, sudden cardiac death, and stroke for every 1-mg/dL increase in phosphate.^20^ A large cohort study in HD observed an increased risk of cardiovascular related hospitalizations in those with a serum phosphate level between 1.61 and 2.90 mmol/L compared to those with lower levels. Importantly, future randomized controlled trials will test whether changing phosphate levels will reduce the risk of cardiovascular morbidity and mortality. For example, the Pragmatic Randomised Trial of High or Standard Phosphate Targets in End-Staged Kidney Disease trial is testing whether intensive lowering of phosphate toward the normal level (≤1.50 mmol/L or 4.65 mg/dL) versus liberal control (2.0-2.5 mmol/L or 6.2-7.75 mg/dL) reduces the risk of fatal or nonfatal myocardial infarction in patients receiving HD. The HiLo trial examined whether a high serum phosphate level (2.1 mmol/L or ≥6.5 mg/dL) versus a lower phosphate level (<1.78 mmol/L or <5.5 mg/dL) is associated with all-cause mortality and hospitalization.^21^

Another highlight of this study is the possible relationship between total ALP and MACE. In observational cohort studies in predialysis and dialyses, those in the highest quartile of ALP also had a higher risk of all-cause mortality.^22^ There has been some some suggestion that lowering ALP may be beneficial for cardiovascular health.^23^

We also observed a mild protective relationship between higher calcium and phosphate levels on fragility fracture. It is known that lower calcium and phosphate levels can lead to osteomalacia and fracture,^24^ so, perhaps, this is an explanation. A large prospective cohort study in HD (n = 6,797) observed that a baseline serum phosphate level of >1.97 mmol/L or 6.1 mg/dL was associated with a higher risk of fracture and so there is likely an upper threshold for both phosphate and calcium. Devoted studies of the optimal range are important.^25^ Regarding the observed relationship between ALP and fracture, like in our study, a small observational cohort study of those receiving HD, noted that fragility fractures occurred in 21 (15.1%) over 2 years, and the fracture rates were significantly higher when total ALP levels were higher.^26^ Observational studies have also suggested that total ALP may have some value in predicting fracture in those receiving dialysis.^26^

Our study findings also corroborate with other reports, that have observed a U-shaped relationship between PTH and fragility fracture.^17^^,^^18^ There is cortical thinning, increased cortical porosity, and trabecularization of the endocortical bone when PTH is high, and the adynamic bone when PTH is low.^17^^,^^18^ A recent study in dialysis noted a lower PTH level to actually be protective - there was an odds ratio of fracture per intact PTH doubling of 1.06 (95% confidence interval 1.03-1.09) which was most pronounced for hip fracture.^27^ On the balance, a high bone turnover (ie, high PTH and ALP) appears consistently detrimental to bone per our study and the literature.

The strengths of our study include its large sample size, contemporary time frame, and the availability of laboratory data from across the province of Ontario. We highlight gaps in the monitoring and control of CKD-MBD and note that calcium levels at the higher end of normal, ALP in the lower end of normal, normal phosphate, and PTH between 3X and 10X the upper limit of normal might be best for bone and heart health. That said, we recognize our study limitations including its generalizability to our province only, and our lack of ability to examine interrelationships between laboratory tests including hyperparathyroidism and low 25 OHD owing to limited events. PTH values do range by laboratory, and to try to harmonize these, used 2X-9X the upper normal limit for the laboratory test center as our target. Moreover, we recognize that sociodemographic factors might influence care patterns (eg, income, race, and ethnicity), but examining the predictors of suboptimal care was outside the scope of our study. It is also important to note that CKD can progress over time and so for inclusion into our cohort, we required individuals to have stable kidney function. We also chose a relatively short follow-up time (ie, 365 days) to minimize the chance of significant changes in kidney function with time. In prior studies in our jurisdiction, we have found that even a single outpatient creatinine level is usually stable and can be representative of a patient’s chronic kidney function.^28^ Although we did explore the association between CKD-MBD and clinically important outcomes as exploratory analyses, examining these relationships was not the primary intent of this study. Future studies could be designed to examine associations using a larger cohort.

In conclusion, there may be opportunities to improve the monitoring and control of CKD-MBD in patients with CKD and end-stage kidney disease. Given the link between CKD-MBD and the clinical outcomes observed in this study and others, we might refocus on this disease and its management.
