# Supplementary material for: Monitoring, Control, and Clinical Outcomes Associated With Chronic Kidney Disease-Mineral Bone Disorder: A Population-Based Cohort Study in Ontario, Canada
Source: Kidney Med. 2025 Aug 6;7(10):101080. doi: 10.1016/j.xkme.2025.101080 (PMC12509893; doi:10.1016/j.xkme.2025.101080)
Supplement: Supplementary File (PDF) — Figures S1, S2; Tables S1-S10. [file mmc1.pdf]

Figure S1. Association between PTH and fracture in those with stage 4 and 5 CKD using restricted cubic splines. Knots were placed at the 5th, 25th, 50th, 75th and 95th percentiles

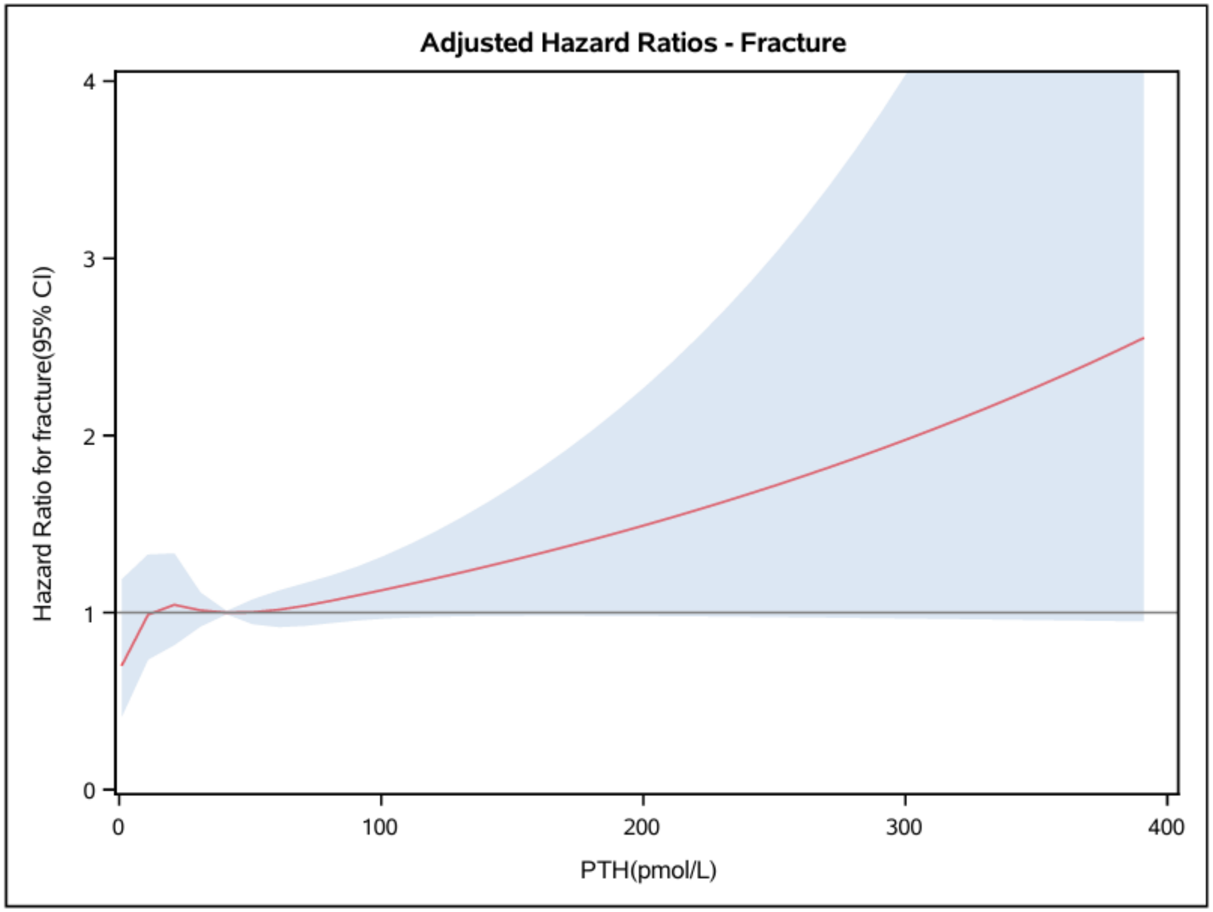

Figure S2. Association between PTH and fracture in those receiving maintenance dialysis using restricted cubic splines. Knots were placed at the 5th, 25th, 50th, 75th and 95th percentiles

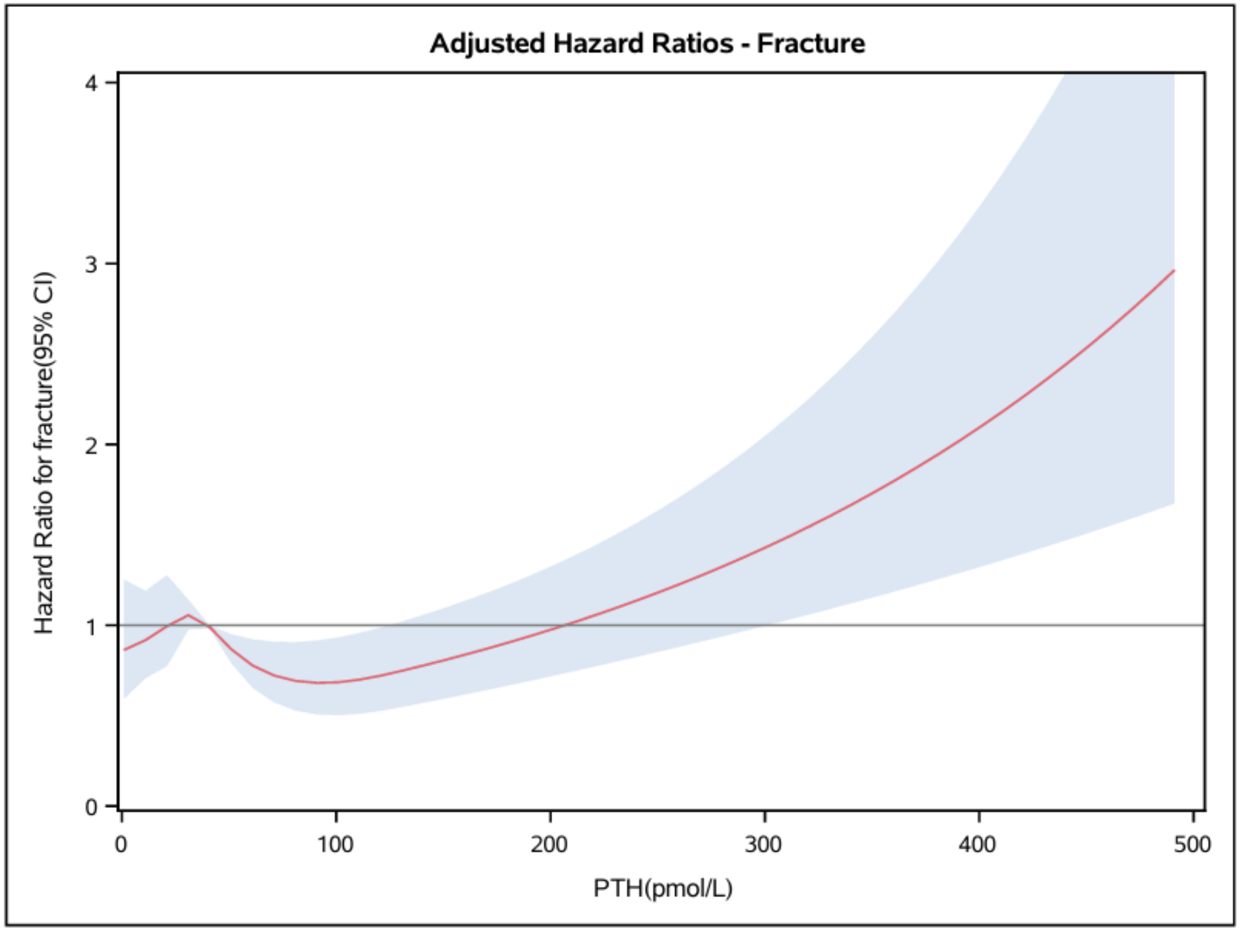

Table S1. RECORD statement

		**STROBE items**	**RECORD items**	**Location in manuscript where items are reported**
	1	(a) Indicate the study's design with a commonly used term in the title or the abstract (b) Provide in the abstract an informative and balanced summary of what was done and what was found	RECORD 1.1: The type of data used should be specified in the title or abstract. When possible, the name of the databases used should be included.<br><br>RECORD 1.2: If applicable, the geographic region and timeframe within which the study took place should be reported in the title or abstract.<br><br>RECORD 1.3: If linkage between databases was conducted for the study, this should be clearly stated in the title or abstract.	Title page
Background rationale	2	Explain the scientific background and rationale for the investigation being reported		Introduction
Objectives	3	State specific objectives, including any prespecified hypotheses		Introduction
Study Design	4	Present key elements of study design early in the paper		Methods
Setting	5	Describe the setting, locations, and relevant dates, including periods of recruitment, exposure, follow-up, and data collection		Methods
Participants	6	*(a) Cohort study* - Give the eligibility criteria, and the sources and methods of selection of participants. Describe methods of follow-up<br>*Case-control study* - Give the eligibility criteria, and the sources and methods of case ascertainment and control selection. Give the rationale for the choice of cases and controls	RECORD 6.1: The methods of study population selection (such as codes or algorithms used to identify subjects) should be listed in detail. If this is not possible, an explanation should be provided.<br><br>RECORD 6.2: Any validation studies of the codes or algorithms used to select the population should be referenced. If validation was conducted for this study and not published elsewhere, detailed methods and results	Methods<br>Appendix

		Cross-sectional study - Give the eligibility criteria, and the sources and methods of selection of participants<br><br>*(b) Cohort study* - For matched studies, give matching criteria and number of exposed and unexposed<br>*Case-control study* - For matched studies, give matching criteria and the number of controls per case	should be provided.<br><br>RECORD 6.3: If the study involved linkage of databases, consider use of a flow diagram or other graphical display to demonstrate the data linkage process, including the number of individuals with linked data at each stage.	
Variables	7	Clearly define all outcomes, exposures, predictors, potential confounders, and effect modifiers. Give diagnostic criteria, if applicable.	RECORD 7.1: A complete list of codes and algorithms used to classify exposures, outcomes, confounders, and effect modifiers should be provided. If these cannot be reported, an explanation should be provided.	Appendix
Data sources/ measurement	8	For each variable of interest, give sources of data and details of methods of assessment (measurement).<br>Describe comparability of assessment methods if there is more than one group		Methods
Bias	9	Describe any efforts to address potential sources of bias		Methods
Study size	10	Explain how the study size was arrived at		Results
Quantitative variables	11	Explain how quantitative variables were handled in the analyses. If applicable, describe which groupings were chosen, and why		Methods, Table 1, Supplement
Statistical methods	12	(a) Describe all statistical methods, including those used to control for confounding<br>(b) Describe any methods used to examine subgroups and interactions<br>(c) Explain how missing data were addressed<br>(d) *Cohort study* - If applicable, explain how loss to follow-up was addressed<br>*Case-control study* - If applicable, explain how matching of cases and controls was addressed		Methods

		Cross-sectional study - If applicable, describe analytical methods taking account of sampling strategy<br>(e) Describe any sensitivity analyses		
Data access and cleaning methods		..	RECORD 12.1: Authors should describe the extent to which the investigators had access to the database population used to create the study population.<br><br>RECORD 12.2: Authors should provide information on the data cleaning methods used in the study.	Methods
Linkage		..	RECORD 12.3: State whether the study included person-level, institutional-level, or other data linkage across two or more databases. The methods of linkage and methods of linkage quality evaluation should be provided.	
Participants	13	(a) Report the numbers of individuals at each stage of the study (*e.g.*, numbers potentially eligible, examined for eligibility, confirmed eligible, included in the study, completing follow-up, and analysed)<br>(b) Give reasons for non-participation at each stage.<br>(c) Consider use of a flow diagram	RECORD 13.1: Describe in detail the selection of the persons included in the study (*i.e.,* study population selection) including filtering based on data quality, data availability and linkage. The selection of included persons can be described in the text and/or by means of the study flow diagram.	Results Supplement Figure 1
Descriptive data	14	(a) Give characteristics of study participants (*e.g.*, demographic, clinical, social) and information on exposures and potential confounders<br>(b) Indicate the number of participants with missing data for each variable of interest<br>(c) *Cohort study* - summarise follow-up time (*e.g.*, average and total amount)		Results, Table 1, Supplement
Outcome data	15	*Cohort study* - Report numbers of outcome events or summary measures over time<br>*Case-control study* - Report numbers in each exposure category, or summary measures of exposure<br>*Cross-sectional study* - Report numbers		Results

		of outcome events or summary measures		
Main results	16	(a) Give unadjusted estimates and, if applicable, confounder-adjusted estimates and their precision (e.g., 95% confidence interval). Make clear which confounders were adjusted for and why they were included<br>(b) Report category boundaries when continuous variables were categorized<br>(c) If relevant, consider translating estimates of relative risk into absolute risk for a meaningful time period		Results, Table 2, Table 3, Figure 1
Other analyses	17	Report other analyses done—e.g., analyses of subgroups and interactions, and sensitivity analyses		Results
Key results	18	Summarise key results with reference to study objectives		Discussion
Limitations	19	Discuss limitations of the study, taking into account sources of potential bias or imprecision. Discuss both direction and magnitude of any potential bias	RECORD 19.1: Discuss the implications of using data that were not created or collected to answer the specific research question(s). Include discussion of misclassification bias, unmeasured confounding, missing data, and changing eligibility over time, as they pertain to the study being reported.	Discussion
Interpretation	20	Give a cautious overall interpretation of results considering objectives, limitations, multiplicity of analyses, results from similar studies, and other relevant evidence		Discussion
Generalisability	21	Discuss the generalisability (external validity) of the study results		Discussion
Funding	22	Give the source of funding and the role of the funders for the present study and, if applicable, for the original study on which the present article is based		Acknowledge ments
Accessibility of protocol, raw data, and programming		..	RECORD 22.1: Authors should provide information on how to access any supplemental information such as the study protocol, raw data, or programming code.	The dataset from this study is held securely in

code				coded form at ICES. While legal data sharing agreements between ICES and data providers (e.g., healthcare organizations and government) prohibit ICES from making the dataset publicly available, access may be granted to those who meet pre-specified criteria for confidential access, available at www.ices.on.ca/DAS (email: das@ices.on.ca). The full dataset creation plan and underlying analytic code are available from the authors upon

				request, understanding that the computer programs may rely upon coding templates or macros that are unique to ICES and are therefore either inaccessible or may require modification.

Table S2. Database Descriptions

Database	Description
**Registered Persons Database**	Provides demographic information for all individuals who have ever received an Ontario health card number. Information includes
**ICES Physicians Database**	Contains information about physicians practicing in Ontario (e.g., demographics, specialty, services provided)
**Corporate Provider Database**	Contains information about clinicians practicing in Ontario
**Ontario Renal Reporting System**	Contains activity data on multi-care kidney clinics, acute dialysis and chronic dialysis provided by kidney care providers
**Discharge Abstract Database**	A dataset created by the Canadian Institute for Health Information and contains administrative, clinical, and demographic information regarding hospital discharges.
**National Ambulatory Care Reporting System**	A dataset maintained by the Canadian Institute for Health Information and includes information regarding hospital and community-based ambulatory care such as day surgery and emergency department visits.
**Ontario Drug Benefits Claims Database**	Identifies drug, dose, and date for outpatient drug dispensations through publicly funded drug programs in Ontario. Eligible recipients include Ontario residents aged 65 and over and select younger populations.
**Ontario Health Insurance Plan Claims Database**	Records all claims for reimbursement by Ontario physicians for inpatient and ambulatory visits, consultations, and procedures
**Same Day Surgery Database**	Records ambulatory care visits for inpatient surgery or to the emergency department.
**Ontario Crohn's and Colitis Cohort Database**	Validated cohort of people with inflammatory bowel disease in Ontario
**Hypertension Dataset**	Validated cohort of individuals with hypertension in Ontario
**Ontario Diabetes Database**	Validated cohort of people with diabetes in Ontario
**Ontario Rheumatoid Arthritis Database**	Validated cohort of people with rheumatoid arthritis in Ontario
**Chronic obstructive pulmonary disease dataset**	Validated cohort of people with COPD in Ontario

**Ontario Asthma Dataset**	Validated cohort of people with asthma in Ontario
**Dementia**	Validated cohort of individuals with dementia in Ontario
**Client Agency Patient Enrolment Database**	Dataset that identified patients enrolled in different primary care models over time.
**Ontario Laboratory Information System Database**	Collects lab results of patients from all Public Health Ontario laboratories and a number of hospitals and community laboratories.
**Ontario Marginalization Index Database**	Dataset assesses socioeconomic vulnerability based on place of residence.
**Drug Identification Database**	Contains information on Canadian drug identification numbers

Table S3. Variable definitions

Characteristic	Data Sources	Variables/Code Types	Look back
Age	RPDB	NA	At Index
Sex	RPDB	NA	Index date
Hypertension	HYPER	DIAGDATE, DIAGDATE_LAST	To earliest available
Diabetes	ODD	DIAG_LAST, DIAGDATE	To earliest available
Renal Transplant	OHIP<br>ORRS	FEECODE<br>TREATMENTCHANGECD	To earliest available
Obesity	CIHI DAD	ICD 10 and ICD-10 CA	5 year lookback
MI	CIHI-DAD<br>OHIP	ICD 10 and ICD-10 CA	5 year lookback
Peripheral Vascular disease	CIHI-DAD<br>OHIP	ICD 10 and ICD-10 CA<br>CCI<br>FEECODE	5 year lookback
CVD	CIHI-DAD<br>OHIP	ICD 10 and ICD-10 CA	5 year lookback
Chronic liver disease	CIHI-DAD<br>OHIP	ICD 10 and ICD-10 CA<br>FEECODEDXCODE	At index
Depression	CIHI-DAD<br>NACRS	ICD 10 and ICD-10 CA	5 year look back

	OHIP		
Rheumatoid Arthritis (RA)	ORAD	DIAGDATE_LAST<br><br>DIAGDATE_FIRST	To earliest available
Psoriasis	CIHI-DAD<br><br>OHIPDX	ICD 10 and ICD-10 CA	5 year look back
Osteoarthritis	CIHI-DAD<br><br>NACRS	ICD 10 and ICD-10 CA	5 year look back
COPD	COPD	DIAGDATE	To earliest available
Asthma	ASTHMA	DIAGSRC<br>FIRSTOHIP	to earliest available
IBD	OCCC		To earliest available
SLE	CIHI- DAD	ICD 10 and ICD-10 CA	5 year look back
Major cancer	OHIP<br><br>CIHI-DAD	ICD 10 and ICD-10 CA	5 year look back
Dementia	DEMENTI<br>A	DIAGDATE<br><br>FIRSTDADSDS<br>FIRSTODB<br>FIRSTOHIP	To earliest available date
Mean number of visits to nephrologist	CPDB<br><br>RPDB<br><br>IPDB<br>OHIP	MAIN PRACTICE SPECIALITY (IPDB)<br><br>DESCRIPTION (CPDB)	365 days prior to and including the index date

Mean number of physician visits to an endocrinologist	CPDB<br>RPDB<br>IPDB<br>OHIP	MAIN PRACTICE SPECIALITY (IPDB)<br>DESCRIPTION (CPDB)	365 days prior to and including the index date
Parathyroidectomy	SDS<br>OHIP<br>CIHI-DAD	ICD 10 and ICD-10 CA	5 year look back

Table S4. Targets for the annual monitoring and control of calcium, PTH, ALP, phosphate, 25 OHD in the maintenance dialysis population

CKD stage	Serum calcium		PTH		ALP		Phosphate		25 OHD	
	Times/ yr	Target value	Times /yr**	Target value	Times /yr	Target value ***	Times/yr	Target value ****	Times /yr *****	Target value
Stage 3	1-2	NA	1-2	NA	1	NA	1-2	NA		NA
Stage 4	2-4						2-4			
Stage 5										
Dialysis	4-12	2.12 to 2.52 mmol/L	2-4	2-9x the upper limit of normal (i.e. 14-63 pmol/L)		50-126 U/L	4-12	0.81 to 1.80 mmol/L		>75 nmol/L

*KDIGO suggests avoidance of hypercalcemia. In this study we chose the referent calcium range for most laboratories.

** KDIGO recommends monitoring PTH based on baseline level, and CKD progression

***No consensus on target. In this study chose referent ALP range for most laboratories

****KDIGO suggests targeting a phosphate toward the upper limit of normal. In this study chose referent phosphate for most laboratories

***** In all stages of CKD guidelines suggest that 25OHD levels might be measured but frequency is based on baseline values. In this study chose lower limit of reference range for most laboratories

Table S5. Mean and median number of lab tests by CKD stage in the 365 days following the index date

Lab test		Stage 1: >90	Stage 2: 60 - <90	Stage 3a: 45-59	Stage 3b: 30 - <45	Stage 4: 15 - <30	Stage 5: <15, not receiving dialysis	Dialysis
		N=1,215,150	N=1,061,747	N=191,761	N=76,452	N=20,699	N=3,765	N=11,207
Ca2+:	Mean (SD)	0.40 (1.56)	0.46 (1.58)	0.71 (1.86)	1.26 (2.24)	2.68 (3.05)	8.31 (8.00)	9.33 (8.01)
	Median (IQR)	0.00 (0.00-0.00)	0.00 (0.00-0.00)	0.00 (0.00-1.00)	1.00 (0.00-2.00)	2.00 (1.00-4.00)	7.00 (3.00-12.00)	9.00 (5.00-12.00)
	No test*	976,981 (80.4%)	823,553 (77.6%)	129,498 (67.5%)	38,033 (49.7%)	4,857 (23.5%)	198 (5.3%)	1,011 (9.0%)
PO43-:	Mean (SD)	0.19 (1.19)	0.22 (1.21)	0.42 (1.50)	0.94 (1.91)	2.35 (2.78)	7.92 (7.42)	8.43 (6.84)
	Median (IQR)	0.00 (0.00-0.00)	0.00 (0.00-0.00)	0.00 (0.00-0.00)	0.00 (0.00-1.00)	2.00 (0.00-3.00)	7.00 (3.00-12.00)	8.00 (4.00-12.00)
	No test	1,111,931 (91.5%)	956,673 (90.1%)	155,221 (80.9%)	46,546 (60.9%)	6,110 (29.5%)	247 (6.6%)	1,100 (9.8%)
ALP:	Mean (SD)	1.04 (1.97)	1.04 (1.95)	1.12 (2.15)	1.24 (2.32)	1.76 (2.80)	5.40 (4.75)	5.99 (5.16)
	Median (IQR)	1.00 (0.00-1.00)	1.00 (0.00-1.00)	1.00 (0.00-1.00)	1.00 (0.00-2.00)	1.00 (0.00-2.00)	4.00 (1.00-9.00)	5.00 (2.00-10.00)
	No test	571,412 (47.0%)	511,291 (48.2%)	94,379 (49.2%)	37,042 (48.5%)	8,470 (40.9%)	576 (15.3%)	1,373 (12.3%)

**PTH:**	Mean (SD)	0.04 (0.27)	0.05 (0.30)	0.13 (0.50)	0.38 (0.88)	1.09 (1.55)	3.08 (2.65)	3.18 (2.54)
	Median (IQR)	0.00 (0.00-0.00)	0.00 (0.00-0.00)	0.00 (0.00-0.00)	0.00 (0.00-0.00)	0.00 (0.00-2.00)	3.00 (1.00-4.00)	3.00 (1.00-5.00)
	No test	1,179,406 (97.1%)	1,025,305 (96.6%)	175,949 (91.8%)	60,280 (78.8%)	11,067 (53.5%)	770 (20.5%)	2,027 (18.1%)
**25(OH)D:**	Mean (SD)	0.16 (0.49)	0.16 (0.49)	0.17 (0.52)	0.21 (0.61)	0.26 (0.71)	0.17 (0.89)	0.23 (0.85)
	Median (IQR)	0.00 (0.00-0.00)	0.00 (0.00-0.00)	0.00 (0.00-0.00)	0.00 (0.00-0.00)	0.00 (0.00-0.00)	0.00 (0.00-0.00)	0.00 (0.00-0.00)
	No test	1,066,486 (87.8%)	929,681 (87.6%)	167,762 (87.5%)	65,392 (85.5%)	17,418 (84.1%)	3,455 (91.8%)	9,958 (88.9%)

*No test was measured in the followup period

Table S6. Mean/median serum value of albumin corrected calcium, phosphate, ALP, PTH and 25 (OHD) by CKD stage

Lab test		Stage 1	Stage 2	Stage 3a	Stage 3b	Stage 4	Stage 5	Dialysis
		N=1,215,150	N=1,061,747	N=191,761	N=76,452	N=20,699	N=3,765	N=11,207
Ca2+:	Mean (SD)	2.22 (0.28)	2.25 (0.27)	2.28 (0.24)	2.30 (0.21)	2.29 (0.21)	2.25 (0.31)	2.27 (0.30)
	Median (IQR)	2.28 (2.22-2.35)	2.30 (2.23-2.37)	2.32 (2.25-2.38)	2.32 (2.25-2.38)	2.30 (2.23-2.38)	2.30 (2.18-2.42)	2.31 (2.20-2.42)
	Mean Contemporary Value (SD):	2.22 (0.30)	2.25 (0.29)	2.28 (0.26)	2.29 (0.23)	2.28 (0.23)	2.25 (0.35)	2.28 (0.33)
	Not calculable*	1,056,453 (86.9%)	895,717 (84.4%)	143,431 (74.8%)	43,328 (56.7%)	5,713 (27.6%)	222 (5.9%)	1,035 (9.2%)
PO43-:	Mean (SD)	1.12 (0.17)	1.10 (0.17)	1.10 (0.18)	1.13 (0.18)	1.22 (0.20)	1.58 (0.39)	1.63 (0.40)
	Median (IQR)	1.12 (1.01-1.24)	1.11 (0.99-1.22)	1.10 (0.99-1.21)	1.13 (1.02-1.24)	1.21 (1.09-1.33)	1.54 (1.32-1.79)	1.59 (1.35-1.86)
	Mean Contemporary Value (SD):	1.12 (0.19)	1.10 (0.19)	1.10 (0.19)	1.13 (0.20)	1.22 (0.24)	1.59 (0.48)	1.64 (0.51)
ALP:	Mean (SD)	76.32 (39.33)	76.27 (37.58)	80.95 (48.65)	86.00 (54.64)	92.19 (60.47)	110.69 (76.67)	112.35 (78.32)
	Median (IQR)	71.00 (59.00-86.00)	71.00 (59.00-86.00)	74.00 (60.50-90.50)	77.00 (63.00-96.00)	82.00 (66.00-104.00)	92.17 (70.89-125.67)	93.15 (72.00-125.67)
	Mean Contemporary Value (SD):	76.65 (46.95)	76.60 (43.13)	81.46 (55.83)	86.54 (60.37)	89.75 (57.16)	112.96 (89.15)	109.38 (87.61)

**PTH:**	Mean (SD)	5.05 (3.26)	5.52 (3.47)	6.61 (4.34)	8.54 (7.64)	13.84 (12.63)	46.77 (51.73)	46.73 (46.05)
	Median (IQR)	4.50 (3.50-5.90)	4.80 (3.68-6.50)	5.65 (4.10-7.85)	7.10 (5.00-10.20)	10.95 (7.20-16.70)	31.30 (16.17-59.61)	34.34 (18.22-59.40)
	Mean Contemporary Value (SD):	4.97 (3.18)	5.45 (3.42)	6.54 (4.36)	8.46 (7.80)	13.87 (13.17)	47.51 (60.74)	47.74 (52.24)
**25 OH D:**	Mean (SD)	74.92 (35.15)	84.43 (35.07)	85.23 (33.97)	82.65 (32.97)	80.51 (33.95)	70.95 (38.93)	63.00 (35.71)
	Median (IQR)	71.00 (52.00-91.50)	81.00 (62.00-101.00)	83.00 (64.00-103.50)	81.00 (61.00-101.00)	80.00 (58.00-99.00)	68.90 (41.00-90.50)	58.00 (35.67-82.50)
	Mean Contemporary Value (SD):	76.42 (36.22)	85.64 (35.69)	86.57 (34.57)	84.31 (33.79)	81.97 (33.97)	70.95 (38.13)	63.71 (36.71)

*Calcium value was not calculable due to missing albumin

Table S7. Percentage of patients receiving dialysis meeting lab targets

Lab test	**Number (n=11,207)**	**%**
Ca2+	6,111	54.5%
PO43-	4,977	39.5%
ALP	6,736	55.6%
PTH*	4,124	37.6%
25(OH)D	361	3.0%
Ca2+ and PO43- and PTH	1,468	13.1%
All	44	0.3%

*Target PTH was defined by a PTH within 2-9 x the upper limit of normal for the lab test centre that the patient visited.

Table S8. Cumulative incidence of fracture and MACE over 2 years of follow-up (n=15 706)

Population	Number of patients	N of events	%	person years of follow-up	Event rate per 1000 person-years	Lower 95% CI	Upper 95% CI
**Fracture**							
**All**	15,706	881	5.6%	24631.0	35.77	33.48	38.21
**Stage 4**	5,287	278	5.3%	9044.4	30.74	27.33	34.57
**Stage 5**	2,802	137	4.9%	3989.4	34.34	29.05	40.60
**Dialysis**	7,617	466	6.1%	11597.3	40.18	36.69	44.00
**MACE**							
**All**	15,706	5,831	37.1%	19660.2	296.59	289.07	304.30
**Stage 4**	5,287	1,646	31.1%	7636.6	215.54	205.38	226.21
**Stage 5**	2,802	967	34.5%	3290.9	293.84	275.89	312.96
**Dialysis**	7,617	3,218	42.2%	8732.7	368.50	355.99	381.45

Table S9. Adjusted association between calcium, phosphate, PTH, ALP, fracture and MACE in CKD 4, 5 and dialysis (n=15, 706)

Outcome	Exposure	Stage 4: 15 - <30 (n=5,287)		Stage 5: <15 (n=2,802)		Dialysis (n=7,617)		Interaction P-value
		HR	95% CI	HR	95% CI	HR	95% CI	
Fracture	Ca (per 0.1 mmol/L)	1.02	0.97-1.07	0.97	0.92-1.01	**0.972**	**0.948-0.998**	0.19
	Phos (per 0.1 mmol/L)	1.02	0.97-1.08	1.00	0.96-1.04	**0.978**	**0.959-0.997**	0.28
	PTH (per 10 pmol/L)	1.04	0.95-1.12	1.027	0.996-1.058	1.010	0.994-1.026	0.58
	ALP (per 10 U/L)	1.017	0.995-1.040	1.013	0.996-1.030	**1.02**	**1.01-1.02**	0.97
MACE	Ca (per 0.1 mmol/L)	1.01	0.99-1.02	1.019	0.999-1.039	1.00	0.99-1.01	0.28
	Phos (per 0.1 mmol/L)	**1.05**	**1.02-1.07**	**1.02**	**1.01-1.04**	**1.01**	**1.00-1.02**	0.07
	PTH (per 10 pmol/L)	1.02	0.99-1.05	1.013	1.000-1.026	0.999	0.992-1.006	0.21
	ALP (per 10 U/L)	**1.012**	**1.003-1.022**	1.003	0.995-1.011	**1.005**	**1.001-1.010**	0.57

Table S10. Adjusted association between calcium, phosphate, PTH, ALP, fracture and MACE in CKD 4, 5 and dialysis (n=15 706)

Outcome	Exposure	HR	95% CI
**Fracture**	Ca (per 0.5 mmol/L)	**0.902**	**0.816-0.998**
	Phos (per 0.5 mmol/L)	0.93	0.86-1.02
	PTH (per 10 pmol/L)	1.01	1.00-1.03
	ALP (per 10 U/L)	**1.02**	**1.01-1.02**
**MACE**	Ca (per 0.5 mmol/L)	1.02	0.98-1.06
	Phos (per 0.5 mmol/L)	**1.09**	**1.06-1.12**
	PTH (per 10 pmol/L)	1.003	0.997-1.009
	ALP (per 10 U/L)	**1.006**	**1.003-1.010**
